# CD11c^+^ T-bet^+^ CD21^hi^ B Cells Are Negatively Associated With Renal Impairment in Systemic Lupus Erythematosus and Act as a Marker for Nephritis Remission

**DOI:** 10.3389/fimmu.2022.892241

**Published:** 2022-05-19

**Authors:** Víctor A. Sosa-Hernández, Sandra Romero-Ramírez, Rodrigo Cervantes-Díaz, Daniel A. Carrillo-Vázquez, Itze C. Navarro-Hernandez, Laura P. Whittall-García, Abdiel Absalón-Aguilar, Ana S. Vargas-Castro, Raúl F. Reyes-Huerta, Guillermo Juárez-Vega, David E. Meza-Sánchez, Vianney Ortiz-Navarrete, Jiram Torres-Ruiz, Nancy R. Mejía-Domínguez, Diana Gómez-Martín, José L. Maravillas-Montero

**Affiliations:** ^1^Red de Apoyo a la Investigación, Instituto Nacional de Ciencias Médicas y Nutrición Salvador Zubirán and Universidad Nacional Autónoma de México, Mexico City, Mexico; ^2^Departamento de Biomedicina Molecular, Centro de Investigación y de Estudios Avanzados del Instituto Politécnico Nacional, Mexico City, Mexico; ^3^Facultad de Medicina, Universidad Nacional Autónoma de México, Mexico City, Mexico; ^4^Departamento de Medicina Interna, Instituto Nacional de Ciencias Médicas y Nutrición Salvador Zubirán, Mexico City, Mexico; ^5^Departamento de Biología Celular, Centro de Investigación y de Estudios Avanzados del Instituto Politécnico Nacional, Mexico City, Mexico; ^6^Departamento de Inmunología y Reumatología, Instituto Nacional de Ciencias Médicas y Nutrición Salvador Zubirán, Mexico City, Mexico

**Keywords:** B cells, aged-associated B cells, systemic lupus erythematosus, lupus nephritis, induction theraphy

## Abstract

Lupus nephritis (LN) is one of the most common manifestations of systemic lupus erythematosus (SLE), characterized by abnormal B cell activation and differentiation to memory or plasma effector cells. However, the role of these cells in the pathogenesis of LN is not fully understood, as well as the effect of induction therapy on B cell subsets, possibly associated with this manifestation, like aged-associated B cells (ABCs). Consequently, we analyzed the molecules defining the ABCs subpopulation (CD11c, T-bet, and CD21) through flow cytometry of blood samples from patients with lupus presenting or not LN, following up a small sub-cohort after six months of induction therapy. The frequency of ABCs resulted higher in LN patients compared to healthy subjects. Unexpectedly, we identified a robust reduction of a CD21^hi^ subset that was almost specific to LN patients. Moreover, several clinical and laboratory lupus features showed strong and significant correlations with this undefined B cell subpopulation. Finally, it was observed that the induction therapy affected not only the frequencies of ABCs and CD21^hi^ subsets but also the phenotype of the CD21^hi^ subset that expressed a higher density of CXCR5. Collectively, our results suggest that ABCs, and more importantly the CD21^hi^ subset, may work to assess therapeutic response since the reduced frequency of CD21^hi^ cells could be associated with the onset of LN.

## Introduction

Systemic lupus erythematosus (SLE) is an autoimmune disorder characterized by a deleterious immune response that affects several tissues and organs, including kidneys. Lupus nephritis (LN) is one of the most common manifestations of this chronic disease, constituting a poor prognosis feature that increases morbidity and mortality (1–3). Over the years, different factors that contribute to LN pathogenesis have been identified being one of the most relevant, the deleterious function of B cells, as it generates hyper-reactive plasma cells that produce autoantibodies that can cause permanent damage within the glomerular, vascular, and tubulo-interstitial compartments of the kidneys, leading to acute or chronic renal failure (4). Nevertheless, the role of B cells in SLE beyond their differentiation to plasma cells and subsequent autoantibodies secretion is still unclear (5).

Analyzing specific circulating B cell subsets to identify alterations in their frequency or phenotype is one of the main approaches that would allow identifying possible roles of this lymphocyte lineage and their specific subpopulations in autoimmune diseases (6). Accordingly, the discovery and characterization of the Aged-associated B Cells (ABCs) subset has been relevant by its association with pro-inflammatory contexts. ABCs have been found numerically altered in conditions of chronic inflammation that encompass autoimmune diseases (SLE, rheumatoid arthritis, systemic sclerosis, etc.) or chronic infection diseases (HIV infection, malaria, hepatitis C virus infection, etc.) (7–10). This rare B cell subset has been defined by the expression of the α-integrin subunit CD11c and the transcription factor T-bet, as well as a low-level expression of the complement receptor type 2 (CR2)/CD21 (11). Regarding their functional properties, ABCs are highly responsive to innate stimuli such as TLR-7 ligands; they may produce inflammatory cytokines (including TNF-α and IFNγ) and block the generation of conventional B cells progenitors by inducing apoptosis through the expression of high levels of surrogate light chain (11–13). Furthermore, upon activation, these cells can rapidly expand, differentiate and produce antibodies (mainly IgG); in fact, higher frequencies of circulating ABCs have been correlated with elevated titers of autoantibodies in patients with SLE (14).

Although ABCs features are particularly well described in mouse models, there are many unsolved questions about their implication in human autoimmune diseases, including SLE, particularly how they are linked to the evolution of the disease, their impact on patients’ treatments, or if alterations in its phenotype could be used as prognostic markers. Consequently, we analyzed the ABCs subset and similar B cell phenotypes in cohorts of SLE patients exhibiting or not LN. We found that patients with LN present low or null frequencies of the atypical “ABC-like” CD11c^+^T-bet^+^CD21^hi^ B cell subset, in contrast with healthy individuals and SLE patients without LN. This subpopulation rendered significant correlations with different features of SLE. Interestingly, both ABCs and CD21^hi^ subsets presented alterations in kidney disease patients after induction therapy, making them attractive candidates to constitute markers that could define patients’ status or prognosis in LN onset.

## Methods

### Patients and Healthy Individuals

We analyzed blood samples from a cohort of 10 patients with SLE without LN (non-LN) in the 5 previous years to recruitment and 17 patients with active LN. They were followed-up in a tertiary care center (Instituto Nacional de Ciencias Médicas y Nutrición Salvador Zubirán in Mexico City, Mexico). All patients without lupus nephritis were confirmed by laboratory and clinical features. All SLE patients fulfilled ACR and SLICC classification criteria for SLE (15, 16). LN was confirmed by a renal biopsy and classified by glomerular disease type using the criteria of the International Society of Nephrology/Renal Pathology Society (ISN/RPS) (17). Notably, the first blood sample of these patients was obtained in a period no longer than three weeks after renal biopsy. Moreover, we included a cohort of 10 age-matched healthy individuals as controls. Exclusion criteria were applied to cohorts with any acute or chronic infection, pregnancy, puerperium, or neoplasia. None of the study participants received any B cell-depleting or other biological therapies. The main demographic and clinical characteristics of all these individuals are depicted in **Table 1**. **Supplementary Table 1** shows that LN patients’ groups, either followed or not followed up, do not display significant baseline differences in their clinical characteristics. Additionally, **Supplementary Table 2** displays the immunosuppressants and dosage administered for induction therapy of LN patients.

**Table 1:** Demographics, clinical and laboratory features of SLE/LN patients.

Features	SLE non-LN	LN month 0	LN month 6
**Gender - #**			
Male	2	7	4
Female	8	10	5
Age in years – median	30 (28-32)	26 (20-33)	22 (20-39)
**Disease Activity - median**			
SLEDAI score (min.-max.)	2 (0-6)	22 (16-35)	8 (2-12)
**Laboratory Values - median**			
White blood cell count/mL [10^6^]	5.4 (4.1-5.82)	6.5 (5.45-9.75)	8.0 (4.7-8.8)
Absolute lymphocyte count/mL [10^6^]	1.32 (1.00-1.62)	0.75 (0.48-1.07)	1.12 (0.70-1.74)
Monocytes, %	7.3 (6.8-8.4)	6.3 (4.6-8.8)	8.7 (7.6-9.5)
Neutrophils, %	62.5 (50.7-71.8)	81.1 (74.8-89.1)	72 (64.9-78.9)
Platelet count, K/µL	205 (180-269)	167 (91.5-228)	271 (214-326)
B cells, %	14.7 (10.91-23.78)	22.3 (17.75-24.95)	5.4 (5.05-8.69)
Creatinine, mg/dL	0.6 (0.5-0.7)	2.2 (1.4-3.9)	0.9 (0.6-1.1)
eGFR, mL/min	119 (112-159)	35.4 (27.9-54.8)	107 (74-127)
C3, mg/dL	110 (88.0-118.8)	53 (40.5-60.5)	99 (90.5-120.5)
C4, mg/dL	23.5 (16.7-26.7)	8 (8-12.5)	30 (14.5-38.5)
Anti-dsDNA (UI/mL)	30 (8.3-62.0)	481 (128.1-688.1)	7.9 (4.4-56.9)
**Treatments – #**			
Mycophenolate Mofetil	1	8	5
Cyclophosphamide	–	9	4
Prednisone	2	17	9
Hydroxychloroquine	6	10	6
Chloroquine	1	4	2
Azathioprine	2	1	–
**Classification of Lupus Nephritis by ISN/RPS**			
Class IV	–	2	1
Class III+V	–	2	1
Class IV+V	–	13	7
**Outcomes - #**			
No Remission			2
Partial Remission	–	–	4
Complete Remission	–	–	3
**Healthy individuals**			
Male	4		
Female	5		
Age in years – median	28 (25-35)		
White blood cell count/mL [10^6^]	4.2 (4.1-5.1)		
Absolute lymphocyte count/mL [10^6^]	0.81 (0.64-0.92)		
B cells, %	7.6 (5.04-11.25)		

Data presented are the median (IQR), except for SLEDAI score presented as median (min.-max. values). # represents the number of individuals.

All recruited patients and healthy individuals signed informed consent before their inclusion. The institutional ethics and research committees of the Instituto Nacional de Ciencias Médicas y Nutrición Salvador Zubirán approved the study (Ref. 2555) in compliance with the Helsinki declaration.

### Multiparametric Flow Cytometry Analysis

Peripheral blood mononuclear cells (PBMCs) were isolated by density gradients with Ficoll-Paque (GE Healthcare Life Sciences). Recovered cells were resuspended in RPMI-1640 with phenol red (Gibco), counted, then washed in Cell Staining Buffer (BioLegend) and treated with a human FcX blocker antibody mix (BioLegend^®^) for 10 minutes and performed the viability staining using Zombie UV (BioLegend) according to manufacturer’s instructions. Cells were then immediately stained with the following conjugated monoclonal antibodies: anti-human CD19 Pacific Blue (BioLegend), CD11c PE (BioLegend), CD21 APC-Fire (BioLegend), CD183/CXCR3 BV605 (BioLegend), CD185/CXCR5 APC (BioLegend), CD197/CCR7 PE-Dazzle 594 (BioLegend) for cell surface detection and anti-T-bet BV711 (BioLegend) for intracellular detection. For surface staining, cells were incubated for 30 minutes at 4°C with the antibody cocktail. After washing the cells, we followed the protocol of True-Nuclear^™^ Transcription Buffer Set (BioLegend). We then fixed samples with True-Nuclear^™^ 1X fix buffer and incubated them at room temperature in the dark for 60 minutes. Later, we washed cells two times with True-Nuclear^™^ 1X perm buffer at 1,500 rpm for 5 minutes, then resuspended the cell pellet in True-Nuclear^™^ 1X Perm buffer and added the conjugated monoclonal antibody anti-T-bet (BioLegend) and incubated at room temperature in the dark for 30 minutes. Lastly, cells were washed once with cell staining buffer (BioLegend) and then resuspended in 300 μL of the same buffer for immediate flow cytometric analysis on a BD LSRFortessa using FACSDiva software (BD Biosciences).

**Figure 1:**
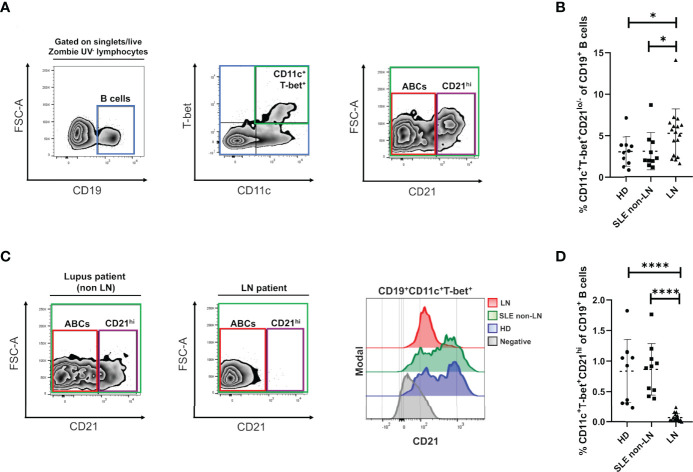
Alterations of ABCs and CD21^hi^ B cell subsets in lupus nephritis. **(A)** Gating strategy for the identification of the indicated B cell subsets in PBMCs, selected from singlets (FSC-A vs. FSC-H), lymphocytes (SSC-A vs. FSC-A) and live Zombie UV^-^ gates. We then selected the CD19 positive cells, to gate over the double positive cells for T-bet and CD11c. Lastly, we segregate these cells by CD21 expression into CD21^lo/-^ (ABCs) and CD21^hi^. To depict this strategy, we present the data obtained from a representative healthy control. **(B)** Comparative analysis of ABCs frequencies (relative to CD19^+^ B cells) between cohorts of healthy donors (HD), non-LN lupus patients and LN. **(C)** Left: representative zebra plots from a non-LN vs. a LN patient to show lack of the CD21^hi^ subset. Right: representative histograms to evaluate density expression of CD21 over the CD19^+^ T-bet^+^ CD11c^+^ cells. **(D)** Comparative analysis of CD21^hi^ frequencies (relative to CD19^+^ B cells) between the same cohorts. All comparative analysis were assessed by a Kruskal-Wallis test followed by a Dunn’s *post-hoc* test. *p ≤ 0.05, ****p ≤ 0.0001.

Up to 1x10^6^ events (cells) were analyzed using FlowJo v10 software (BD Biosciences) with the strategy shown in **Figure 1A**, developed by using Fluorescence Minus One (FMO) controls to define gates plus CompBeads (BD Biosciences) and single stained fluorescent samples to achieve compensation.

### Statistical Analysis

Statistical differences in B cell subsets frequencies between cohorts were assessed by Kruskal-Wallis, followed by Dunn’s *post-hoc* and the Wilcoxon tests. Statistical differences in baseline characteristics between subgroups of LN patients were evaluated by a U-Mann-Whitney test. Comparative analyses of ABCs and CD21^hi^ cell counts regarding employed therapies in LN patients were also performed by a U-Mann-Whitney test. Correlation between B cell subsets frequencies and clinical or laboratory features were evaluated with Spearman’s test. Finally, correlations between ABCs or CD21^hi^ cell absolute numbers with the dosage of induction therapy in LN patients were assessed with Spearman’s test. Prism 9 (GraphPad) and R, v. 4.0.2 (R Foundation for Statistical Computing, Vienna, Austria; URL http://www.R-project.org/) were used to analyze and graph all data sets.

## Results

### The Frequencies of ABC Subset Are Increased in Patients With LN

To understand the alterations of ABCs and their phenotype in the context of LN and patients’ response to induction therapy, we determined these cells’ frequency in peripheral blood. As mentioned before, we discriminated two CD19^+^ cell subpopulations of interest, both expressing CD11c^+^, and T-bet^+^ but segregated by their differential expression of CR2 (CD21), thus defining a CD21^-/lo^ subset (or “classical” ABCs) and a CD21^hi^ subset (**Figure 1A**) that were analyzed in three different groups: healthy individuals, patients with SLE (non-LN) and LN patients. As expected, we observed a notably increase of ABCs frequencies in patients with LN compared to healthy individuals and non-LN patients. However, healthy individuals and patients without LN, do not exhibited a significant difference between them (**Figure 1B**). These observations are supported when absolute cell numbers are assessed since ABCs show the same increase trend in LN patients (**Supplementary Figure 1A**).

### High Expression of CD21 Defines a Different CD11c^+^ T-bet^+^ B Cell Subset That Is Almost Absent in LN Patients

Interestingly, using our flow cytometry approach, we observed a B cell subset that displays a CD11c^+^ and T-bet^+^ phenotype like ABCs but highly expresses the CD21 marker. We detected this CD21^hi^ subset in healthy individuals (**Figure 1A**) and lupus patients without nephropathy (**Figure 1C**, left panel) but was almost absent in LN (**Figure 1C**, middle panel) as shown in the corresponding representative plots. This observation is better shown through a representative histogram (**Figure 1C**, right panel), revealing that healthy individuals and non-LN lupus patients expressed a higher density of CD21 among CD11c^+^ T-bet^+^ B cells compared to nephropathy patients. The healthy subjects and lupus patients without nephropathy groups showed almost the same frequency mean values when all subjects were compared. In contrast, the group of LN patients displays very significant lower CD21^hi^ cell frequencies (**Figure 1D**). Again, if absolute counts are measured, CD21^hi^ cells numbers are significantly reduced in LN patients compared to the other study groups (**Supplementary Figure 1B**).

### The CD21^hi^ B Cell Subset Correlates With Clinical and Laboratory Features of Disease Activity

**Figure 2:**
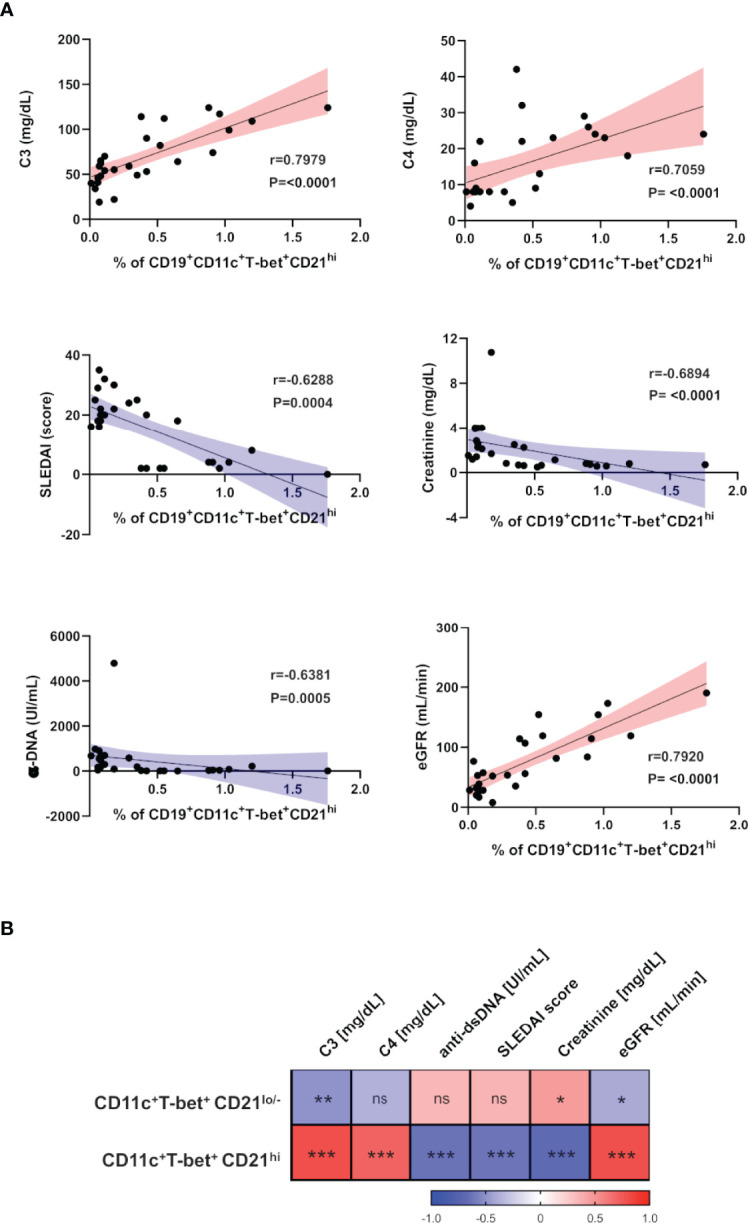
ABCs and CD21^hi^ B cell subsets correlate with different clinical and laboratory parameters in SLE. **(A)** Correlation analysis between peripheral CD21^hi^ subset and levels of C3/C4, SLEDAI score, α-dsDNA, serum creatinine and estimated-GFR. The blue slopes (gradient) present negative correlations and red ones represent positive correlations. All graphs show calculated Spearman’s coefficient (r) and p values (all significant). **(B)** Correlation matrix showing a graphical representation of calculated Spearman’s coefficient calculations between the B cell subset frequencies and clinical and laboratory variables of non-LN patients (n=10) and LN patients before the induction therapy (n=17). The underlying color scale indicates Spearman’s coefficient values. ns, not statistically significant. *p ≤ 0.05 **p ≤ 0.01 ***p ≤ 0.001.

Trying to understand the relevance of the CD21^hi^ subset, we performed correlation analysis between these cell frequencies of non-LN/LN patients and clinical/laboratory features typically assessed in lupus patients. Interestingly, these cells showed a positive correlation with levels of complement (C3/C4) and estimated glomerular filtration rate (eGFR). In contrast, the SLE disease activity index (SLEDAI), titer of antibodies anti-dsDNA and serum creatinine concentration showed a negative correlation (**Figure 2A** and **Table 2**). In addition, we developed a correlation matrix with both subsets, in order to compare their clinical profile. We observed an opposed correlation pattern for these B cell subsets and remarkably, we noticed that the CD21^hi^ subset displayed robust and highly significant correlations, better than those shown by ABCs (**Figure 2B** and **Table 2**).

**Table 2:** Correlations between ABCs or CD21^hi^ cell frequencies with clinical/laboratory features of SLE/LN patients.

Features	ABCs subset		CD21^hi^ subset	
Correlation Values	r	p	r	p
SLEDAI score	0.3576	0.0671	-0.6288	0.0004
Creatinine, mg/dL	0.4624	0.0152	-0.6894	<0.0001
eGFR, ml/min	-0.4045	0.0364	0.7920	<0.0001
C3, mg/dL	-0.5002	0.0079	0.7979	<0.0001
C4, mg/dL	-0.3756	0.0535	0.7059	<0.0001
Anti-dsDNA (UI/mL)	0.3436	0.0856	-0.6381	0.0005

### ABCs Subset Decreases While CD21^hi^ Subset Expands After Induction Therapy

**Figure 3:**
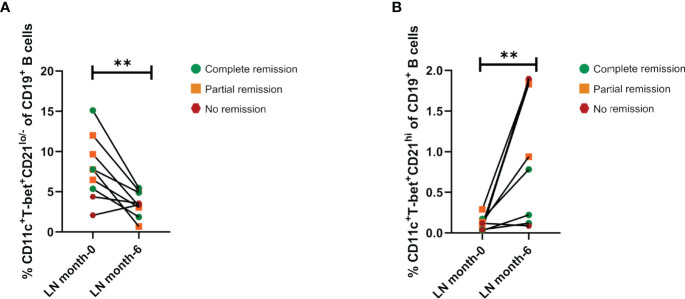
Effect of induction therapy over frequencies of ABCs and CD21^hi^ subsets. **(A)** Comparative analysis of ABCs frequencies (relative to CD19^+^ B cells) between patients at the beginning of induction therapy (month 0), and the same patients after 6 months of treatment (n=9). **(B)** Comparative analysis of CD21^hi^ cell frequencies between patients at the beginning of induction therapy and the same patients after 6 months of treatment (n=9). Both comparative analyses were assessed by a Wilcoxon test. **p ≤ 0.01.

To assess the effects of induction therapy on the ABCs and CD21^hi^ compartments, their frequencies were analyzed again after 6 months of treatment. Since this study was performed during COVID19 pandemic, we were only able to follow up nine LN patients from the total cohort: three of them showed complete remission, four patients exhibited partial remission and two did not show response to treatment, according to guidelines of the European League Against Rheumatism (EULAR) and European Renal Association–European Dialysis and Transplant Association (ERA-EDTA) (2). Although induction therapy does not specifically target B cells, the frequency of ABCs in both complete and partial remission patients exhibit a decrease in this subset, however, one of the patients who showed no response indeed displayed an increase while the other a slight decrease in contrast to the patients who responded to treatment (**Figure 3A**). On the other hand, patients who exhibited partial or complete remission shows an increase in the CD21^hi^ subset (**Figure 3B**). Both ABCs (**Supplementary Figure 2A**) and CD21^hi^ (**Supplementary Figure 2B**) absolute cell numbers were also addressed to confirm our observations, showing the same pattern.

### The CD21^hi^ Subset Is Characterized by a Higher CXCR5 Expression Than the ABCs Subset

**Figure 4:**
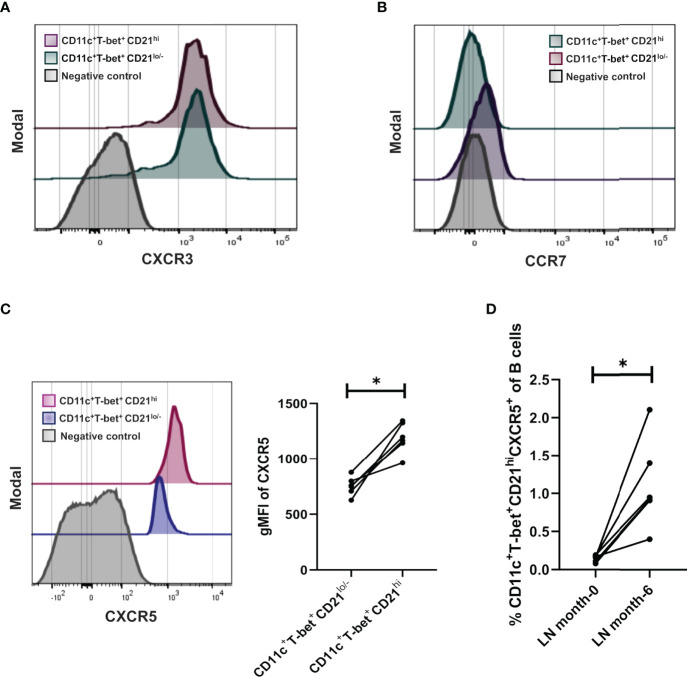
Density expression of chemokine receptors CXCR3, CCR7 and frequencies of CXCR5 in the CD21^hi^ subset. **(A)** Representative histogram of treated LN patients (n=7 responders) to evaluate density expression of CXCR3 between ABCs and CD21^hi^ subsets. **(B)** Representative histogram of treated LN patients to evaluate density expression of CCR7 between ABCs and CD21^hi^ subsets. **(C)** Representative histogram of a treated LN patient to evaluate density expression of CXCR5 between ABCs and CD21^hi^ subsets; further comparative analysis of CD21 gMFI of the mentioned subsets. **(D)** Comparative analysis of CXCR5 frequencies over the CD21^hi^ subset between patients at the beginning of induction therapy and the same patients after 6 months treated. All comparative analysis were assessed by a Wilcoxon test. *p ≤ 0.05.

In order to further characterize the CD21^hi^ subset, we measured the expression of three chemokine receptors in patients who responded to treatment (n= 7 patients). We analyzed CXCR3, CCR7, and CXCR5, which play an essential role in B cell migration in inflammatory and homeostatic conditions (18, 19). CXCR3 expression was present in both subsets and exhibited similar levels (**Figure 4A**). In contrast, CCR7 expression was detected neither in ABCs nor CD21^hi^ subset (**Figure 4B**). Interestingly, the expression of CXCR5 was significantly higher in the CD21^hi^ subset than the ABCs subset in all patients (**Figure 4C**). Besides this, it was evident that samples from untreated (before induction) LN patients presented very low frequencies of CXCR5^+^ CD21^hi^, in contrast with those taken from the same patients after they reach complete or partial remission (**Figure 4D**).

## Discussion

One of the main factors contributing to SLE development and its clinical manifestations are B cells. Several reports concerning these cells have shown their functional, phenotypic, or activation-related alterations. Therefore, the analyses of different B cell subsets have been useful for understanding the pathogenesis of SLE; among them, the T-bet-expressing ABCs represent one of the most intriguing examples. Previous studies in infectious or immune-mediated diseases have evaluated the T-bet transcription factor, integrin CD11c and complement receptor CD21 in these B cells, as molecules associated with aberrant cell activity. However, the relevance of these cells in the pathological, clinical, and therapeutic contexts during autoimmune disorders such as SLE is still not fully clarified.

According to our analysis of ABCs subset, the presence of these cells was detected in our three study groups. In this regard, it has been previously reported that ABCs are increased in lupus patients compared to healthy controls, with a significant difference even more marked when they develop LN (14, 20, 21). As mentioned, our cohorts do not to display that trend, as we could not detect any difference among healthy subjects and lupus patients without active lupus nephritis. Additionally, the LN group of patients only showed a moderate significant increase compared to healthy donors but no difference with non-LN lupus patients. Of course, this trend could be the result of the limited number of recruited patients in our study but also, it could suggest that ABCs are not necessarily as robust for segregating SLE and LN outcomes. Another possible explanation could be that non active disease is associated with absence of a proinflammatory state that could be the main trigger for the increase in ABCs subpopulation.

Prior reports aimed to understand the abnormal activity of B lymphocytes in lupus have detected alterations of ABCs in peripheral blood. The first report to describe a disruption in a similar B cell compartment described an incremented frequency of a CD19^+^ CD21^lo/-^ B cell subset in patients with common variable immunodeficiency (CVID) and SLE (22, 23). Upon this observation, several reports emerged describing and expanding this cell phenotype (including its T-bet expression), besides its association with different chronic infectious diseases and autoimmune disorders (7–10). Despite that, conflicting data about B cells’ CD21 expression in autoimmunity contexts have gone unnoticed. One of the few related approaches was reported by Dash R. et al., which detected an increase in *CR2* (CD21) transcript levels in PBMCs of rheumatoid arthritis patients that negatively correlates with disease activity (24). Regarding SLE, a decrease in the frequency of total CD19^+^ CD21^+^ B cells was previously reported (25). In our present report, the absence of the CD21^hi^ subset in LN patients contrasts with healthy individuals and non-LN patients. Accordingly, the importance of the analysis of CD21 expression can be highlighted. Considering that one of the most important features to development of SLE/LN is the abnormal function of B cells, the integration of these atypical CD21^hi^ B cells to the whole disease landscape, could be relevant to understand phenotypic and functional changes of this cell lineage, thus maybe associated with LN progression.

ABCs have also been proposed as a good marker when following SLE patients since their numbers correlate with some clinical and laboratory features, but mainly with auto-antibody titers (14). These findings also showed that the higher frequency of ABCs implies, in most cases, an increase in the activity of the disease (14, 21). Nevertheless, these correlations are weak or not as significant as those detected in the present study for the CD21^hi^ subpopulation that seems to be more associated to an immunological state of homeostasis.

Beyond the mentioned, there are few descriptions about the effects of induction therapy in LN over B cells; some findings have shown decreased frequencies of plasmablasts/plasma cells associated with mycophenolate mofetil (MMF) administration or the selective depletion of naïve B cells in patients treated with cyclophosphamide (CYC), with little or no effect on class-switched memory B cells in both cases (5). More recently, the effects of the immunosuppressive treatment over a IgD^-^ CD27^-^ B cell subpopulation (double negative; DN) were documented in SLE patients with kidney damage, demonstrating a decreased frequency of these cells in patients who responded to therapy (26). To our knowledge, this is the first study about the effect of induction therapy on ABCs or similar B cell phenotypes. Trying to explain the mechanisms involved in ABCs decrease after the immunosuppressive treatment, we could mention the possible role of IL-21: a cytokine that supports the proliferation of this subset (14, 17). Since MMF therapy has been associated with the inhibition of STAT3 phosphorylation (27) and this transcription factor is linked to the stimulation pathway mediated by IL-21, it would not be surprising that MMF could be directly affecting the functionality of ABCs. However, when we analyzed the potential effects of the different immunosuppressants (MMF or CYC) or dosages employed for induction therapy of followed up LN patients in ABCs or CD21^hi^ subsets, we could not detect any significant shift in their absolute counts regarding the administered drugs (**Supplementary Table 3**) nor any significant correlation between these cell numbers and dosage of the same treatments (**Supplementary Table 4**). Therefore, we hypothesize that additional yet unknown factors, independent of the influence of therapeutics, could contribute to numeric changes in these specific B cell subsets in circulation.

On the other hand, the recovery of CD21^hi^ subset, regardless of a direct effect of induction therapy, could be due to a decrease of different CD21 ligands in circulation after treatment, that includes molecules such as IFN-α, DNA, and C3d, which are classically associated with the physiopathology of SLE (28–30). At this point, we cannot discard that CD21^hi^ cells could not represent an independent B cell subset, thus maybe constituting a transitory stage derived from ABCs. However, as we still do not understand the biological function of this subset, many questions about their identity or origins remain.

To gain insight about functional roles of these cells and considering that the expression of chemoreceptors plays an important role in the activation and maturation of B cells, we evaluated the expression of CXCR3, CCR7 and CXCR5 associated with extra-follicular/follicular pathway, to characterize the putative activation/effector sites of the CD21^hi^ population. An increase in B cells’ CXCR5 expression has been demonstrated when high levels of serum CXCL13 are detected in patients with SLE and nephropathy (31–33). Conflictingly, it has been stated that higher frequencies of CXCR5^-^ CXCR3^+^ B cells correlate with active SLE, besides the presence of CXCR3^+^ B cells in human kidney tissue (18, 34, 35). Since all these reports do not perform extensive phenotyping of these cells, it is possible that our subsets of interest can be overlapped. As ABCs and the recently defined DN subset DN2 (ABCs-like phenotype) have been characterized by a high expression of CXCR3 in contrast to CXCR5 (10, 14, 21), our results regarding these cells correspond to those defining a CXCR3^+^ CXCR5^-/lo^ CCR7^-^ phenotype for ABCs. On the other hand, the neglected CD21^hi^ subset that possesses a higher density of CXCR5 and increases in frequency in post-treatment LN patients, would represent those cells mentioned in the CXCL13/CXCR5 axis reports (31–33). Perhaps by responding to CXCL13, these lymphocytes could migrate towards tertiary lymphoid aggregates to exert putative tissue-associated functions; or maybe, this phenotype could be associated with a follicular pathway that these cells need to follow to be adequately immunologically trained as memory precursors. Accordingly, we recognize that our study is currently restricted by the limited phenotyping of these B cell subsets, that will be further deeply characterized by our group to identify a possible effector role that would promote or cease inflammatory responses.

In summary, we confirmed that ABCs increment their frequency in the circulation of LN patients, and although they cannot be discarded as a factor promoting the pathogenesis of this SLE feature, their numerical alterations could not be as robustly related to LN as those from the non-previously described CD11c^+^ T-bet^+^ CD21^hi^ cells that are almost absent when renal manifestations arise. Importantly, this CD21^hi^ subset could be used as a prognostic factor considering that their numbers strongly correlate with less activity of SLE in our cohort. Furthermore, we found that either ABCs or CD21^hi^ B cell subsets could be considered to assess response to induction therapy in LN. Finally, the identity and functional roles that CD21^hi^ cells perform during the LN onset/course must be studied considering that they may represent a divergent subset different from the already studied ABCs.

## Data Availability Statement

The raw data supporting the conclusions of this article will be made available by the authors, without undue reservation.

## Ethics Statement

The studies involving human participants were reviewed and approved by Ethics and Research Committees of the Instituto Nacional de Ciencias Médicas y Nutrición Salvador Zubirán (Ref. 2555). The patients/participants provided their written informed consent to participate in this study.

## Author Contributions

VS-H contributed to the design and performance of experiments, analysis, and interpretation of data. SR-R and RC-D performed experiments and analyzed data. DC-V, IN-H, LW-G, JT-R, RR-H, and DM-S assisted in obtaining, processing and preservation of patient samples. LW-G, AA-A, and AV-C recruited and collected patient data, generated, and organized our clinical database. GJ-V supports on flow cytometry analyses. VS-H and NM-D performed bioinformatics and statistical analyses. VS-H, VO-N, DG-M, and JM-M designed experiments, supervised general work, wrote, and edited the manuscript. All authors contributed to the article and approved the submitted version.

## Funding

VS-H was supported by CONACyT fellowship 756882/CVU 854621. This work was supported by CONACyT [FOSISS A3-S-36875] and UNAM-DGAPA-PAPIIT Program [IN213020 and IN212122] granted to JM-M, and CONACyT [652260] granted to GJ-V.

## Conflict of Interest

The authors declare that the research was conducted in the absence of any commercial or financial relationships that could be construed as a potential conflict of interest.

## Publisher’s Note

All claims expressed in this article are solely those of the authors and do not necessarily represent those of their affiliated organizations, or those of the publisher, the editors and the reviewers. Any product that may be evaluated in this article, or claim that may be made by its manufacturer, is not guaranteed or endorsed by the publisher.

## Abbreviations

ABC’s, Aged-associated B Cells; CR2 (CD21), Complement Receptor 2; DN, Double Negative B cells; FMO, Fluorescence Minus One; Lk, Leukocyte; Lt, Lymphocyte; Nt, Neutrophil; PBMCs, Peripheral blood mononuclear cells; LN, Lupus Nephritis; SLE, Systemic lupus erythematosus; TLR’s, Toll-like receptors; SLEDAI, Systemic Lupus Erythematosus Disease Activity Index; eGFR, estimated Glomerular Filtration Rate; α-dsDNA, auto-antibody anti-double strand DNA; MMF, Mycophenolate Mofetil; CYC, cyclophosphamide; ISN/RPS, International Society of Nephrology/Renal Pathology Society; ACR, American College of Rheumatology; gMFI, geometric Mean Fluorescence Intensity; CVID, Common Variable Immunodeficiency; RA, Rheumatoid Arthritis; HCV, Hepatitis C Virus.
