# Supplementary material for: CD11c+ T-bet+ CD21hi B Cells Are Negatively Associated With Renal Impairment in Systemic Lupus Erythematosus and Act as a Marker for Nephritis Remission
Source: Front Immunol. 2022 May 19;13:892241. doi: 10.3389/fimmu.2022.892241 (PMC9160198; doi:10.3389/fimmu.2022.892241)
Supplement: Supplementary file 1 [file DataSheet_1.pdf]

Supplementary Table 1. Baseline characteristics of LN patients at recruitment time.

Features	Followed up LN patients (n = 9)	Non-followed up LN patients (n = 8)	p value
SLEDAI score	22 (16-32)	22 (18-35)	0.2644
White blood cell count/mL [10^6]	6.4 (5.8-8.9)	6.8 (3.9-10.1)	0.9303
Absolute lymphocyte count/mL [10^6]	0.69 (0.44-1.22)	0.77 (0.42-0.97)	0.6419
Monocytes, %	6.8 (3.9-8.7)	5.5 (4.4-8.9)	0.9517
Neutrophils, %	81.1 (72.7-87.7)	79.1 (75.8-89.7)	0.8723
Platelet count, K/µL	167 (125-260)	172 (71-231)	0.3154
B cells, %	19.1 (17.8-20.8)	20.6 (18.3-23.1)	0.1940
Creatinine, mg/dL	1.6 (1.15-3.95)	2.3 (2.14-3.65)	0.3238
eGFR, mL/min	32 (28-65)	37 (22-55)	0.4668
C3, mg/dL	48 (37-59)	54 (43-64)	0.5487
C4, mg/dL	8 (8-19)	8 (8-8.75)	0.8114
Anti-dsDNA (UI/mL)	657.3 (355.6-835.9)	183.4 (52.68-434.8)	0.1784

Data presented are the median (IQR). P values were obtained by U-Mann-Whitney test.

Supplementary Table 2. Induction therapy dosage of all LN patients.

Therapy (dose)	LN patients (#)
**Mycophenolate Mofetil (MMF)**	
3000 (mg/day)	3
1500 (mg/day)	1
1000 (mg/day)	4
**Cyclophosphamide (CYC)**	
1000 (mg/day)	7
800 (mg/day)	1
600 (mg/day)	1
**Prednisone**	
60 (mg/day)	17

Supplementary Table 3. Comparative analyses of ABCs and $CD21^{hi}$ cell counts regarding employed therapies in followed-up LN (6 months) patients.

Feature	ABCs subset			$CD21^{hi}$ subset		
Therapy	**MMF** n = 5	**CYC** n =4	**p value**	**MMF** n = 5	**CYC** n =4	**p value**
	2082 (1581-6086)	1239 (896.8-3615)	0.2857	700 (409-1139)	514 (65-2082)	0.9000

Data presented are the median of absolute cell counts (cells/mL) of each subset (IQR). MMF: Mycophenolate Mofetil, CYC: Cyclophosphamide. P values were obtained by U-Mann-Whitney test.

Supplementary Table 4. Correlations between ABCs or CD21^{hi} cell counts with dosage of induction therapy in followed-up LN (6 months) patients.

Therapy	ABCs subset		CD21^{hi} subset	
	r	p	r	p
Mycophenolate Mofetil	-0.2887	0.6376	0.5774	0.3081
Cyclophosphamide	0.6325	0.3675	0.6325	0.3675

Supplementary Figure 1

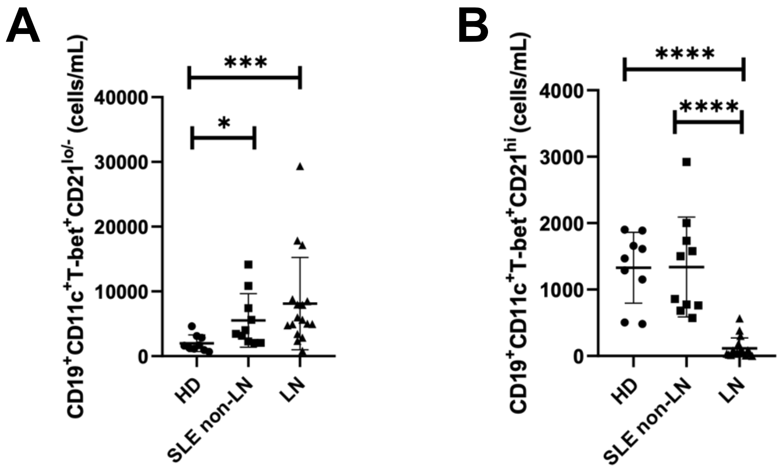

Supplementary Figure 1. Alterations of ABCs and CD21^{hi} B cell subsets absolute counts in lupus nephritis. A) Comparative analysis of ABCs absolute counts between cohorts of healthy donors (HD), non-LN lupus patients and LN. B) Comparative analysis of CD21^{hi} absolute counts between the same cohorts. All comparative analysis were assessed by a Kruskal-Wallis test followed by a Dunn's post-hoc test. *p≤0.05, ***p≤0.001, ****p≤0.0001.

Supplementary Figure 2

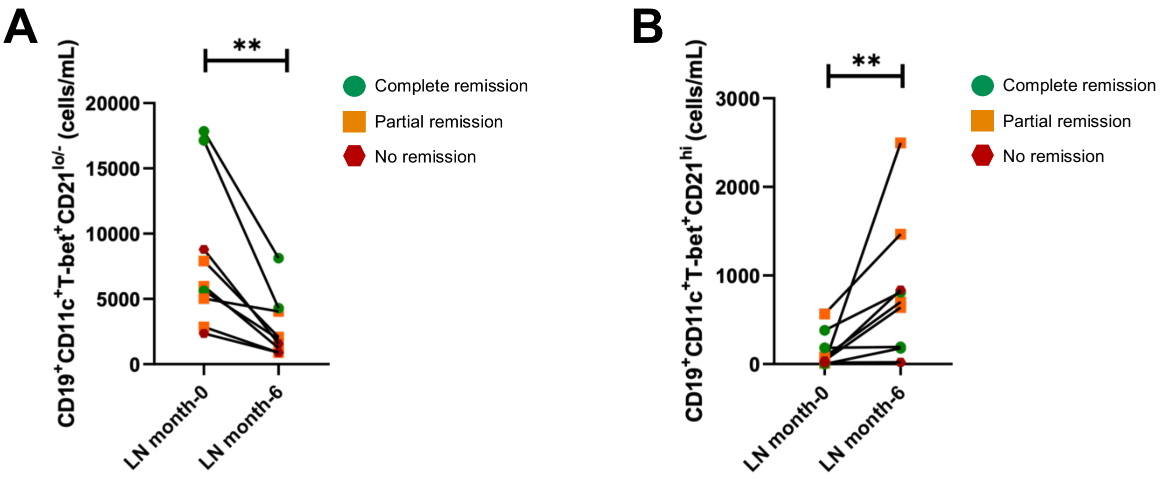

Supplementary Figure 2. Effect of induction therapy over absolute counts of ABCs and CD21^{hi} subsets. A) Comparative analysis of ABCs absolute counts between patients at the beginning of induction therapy (month 0), and the same patients after 6 months of treatment. B) Comparative analysis of CD21^{hi} absolute counts between the same cohorts. Both comparative analyses were assessed by a Wilcoxon test. n = 9, **p≤0.01.
